# Repositioning of Anti-Inflammatory Drugs for the Treatment of Cervical Cancer Sub-Types

**DOI:** 10.3389/fphar.2022.884548

**Published:** 2022-06-13

**Authors:** Medi Kori, Kazim Yalcin Arga, Adil Mardinoglu, Beste Turanli

**Affiliations:** ^1^ Department of Bioengineering, Faculty of Engineering, Marmara University, Istanbul, Turkey; ^2^ Genetic and Metabolic Diseases Research and Investigation Center (GEMHAM), Marmara University, Istanbul, Turkey; ^3^ Science for Life Laboratory, KTH—Royal Institute of Technology, Stockholm, Sweden; ^4^ Centre for Host-Microbiome Interactions, Faculty of Dentistry, Oral and Craniofacial Sciences, King’s College London, London, United Kingdom

**Keywords:** inflammation, drug repurposing, anti-inflammatory drugs, cervical cancer, human papillomavirus 16, human papillomavirus 18

## Abstract

Cervical cancer is the fourth most commonly diagnosed cancer worldwide and, in almost all cases is caused by infection with highly oncogenic Human Papillomaviruses (HPVs). On the other hand, inflammation is one of the hallmarks of cancer research. Here, we focused on inflammatory proteins that classify cervical cancer patients by considering individual differences between cancer patients in contrast to conventional treatments. We repurposed anti-inflammatory drugs for therapy of HPV-16 and HPV-18 infected groups, separately. In this study, we employed systems biology approaches to unveil the diagnostic and treatment options from a precision medicine perspective by delineating differential inflammation-associated biomarkers associated with carcinogenesis for both subtypes. We performed a meta-analysis of cervical cancer-associated transcriptomic datasets considering subtype differences of samples and identified the differentially expressed genes (DEGs). Using gene signature reversal on HPV-16 and HPV-18, we performed both signature- and network-based drug reversal to identify anti-inflammatory drug candidates against inflammation-associated nodes. The anti-inflammatory drug candidates were evaluated using molecular docking to determine the potential of physical interactions between the anti-inflammatory drug and inflammation-associated nodes as drug targets. We proposed 4 novels anti-inflammatory drugs (AS-601245, betamethasone, narciclasin, and methylprednisolone) for the treatment of HPV-16, 3 novel drugs for the treatment of HPV-18 (daphnetin, phenylbutazone, and tiaprofenoic acid), and 5 novel drugs (aldosterone, BMS-345541, etodolac, hydrocortisone, and prednisolone) for the treatment of both subtypes. We proposed anti-inflammatory drug candidates that have the potential to be therapeutic agents for the prevention and/or treatment of cervical cancer.

## Introduction

According to data collected worldwide in 2018, cervical cancer was the fourth most commonly diagnosed cancer (570,000 cases) and the fourth leading cause of death (311,000 deaths) (Bray et al., 2018). In 2020, cervical cancer caused 13,800 new cases and 4,290 deaths in the United States (Siegel et al., 2020). Infection with highly oncogenic Human Papillomaviruses (HPVs) is encountered in almost all cervical cancers (Ferris et al., 2020). HPV is a small, non-enveloped, circular, double-stranded DNA that belongs to the Papillomaviridae family. More than a hundred HPV types with different oncogenic potential (low-risk and high-risk) have been characterized today (Graham, 2010). Of the 12 high-risk HPV types, HPV16 and HPV18, are the most prevalent HPV types worldwide. Indeed, HPV16 and HPV18 are responsible for up to 70% of cervical cancers worldwide (WHO, 2021).

Vaccines are now being proposed to prevent cervical cancer (Šarenac and Mikov, 2019). Various antineoplastic agents such as cisplatin, carboplatin, ifosfamide, paclitaxel, and topotecan have been proposed to treat cervical cancer. However, these antineoplastic agents were not specific to cervical cancer (Ordikhani et al., 2016). Hence, there is a need for the development of more effective prevention and/or treatment strategies to replace the existing methods.

Drug repurposing means identifying new therapeutic purposes for existing drugs. Due to its high efficiency in terms of time saving and low cost compared to traditional approaches for drug development, the pharmaceutical research industry is showing great interest in drug repurposing (Jarada et al., 2020). The inhibitory effects of several drugs, including metformin (Xia et al., 2020), aspirin (Friel et al., 2015), and acetaminophen (Liu et al., 2014), have been demonstrated in cervical cancer. Computational drug repurposing applications for cervical cancer are limited. Recent studies on computational drug repurposing used docking and molecular dynamics simulations to find potential E6 inhibitors in cervical cancer. They suggested valganciclovir and cytarabine as drug candidates and reported ASK4, a valganciclovir derivative, as a potential E6 inhibitor (Kumar et al., 2020).

Inflammation is often described as a response to invasive pathogen simulations. When the inflammatory response is absent or cannot be controlled, it results in impaired tissue repair or pathology. For these reasons, inflammation is now referred to as the seventh hallmark of cancer. It was known that inflammation following viral infection promotes the development of cancer (Deivendran et al., 2014). Studies in recent years have shown that increased dietary intake of native anti-inflammatory compounds (e.g., curcumin) contributes to the prevention of cancer. Furthermore, persistent infection is essential for cancer development, and anti-inflammatory drugs generally target signaling pathways used by oncogenic viruses to generate persistent infections. Therefore, anti-inflammatory drugs may not only reduce the prevalence of oncogenic cancers and but also support ongoing treatment strategies (Read and Douglas, 2014). Taken together, eliminating inflammation may be a valid strategy for cancer prevention and/or treatment, particularly oncogenic cancers (Rayburn et al., 2009).

**FIGURE 1:**
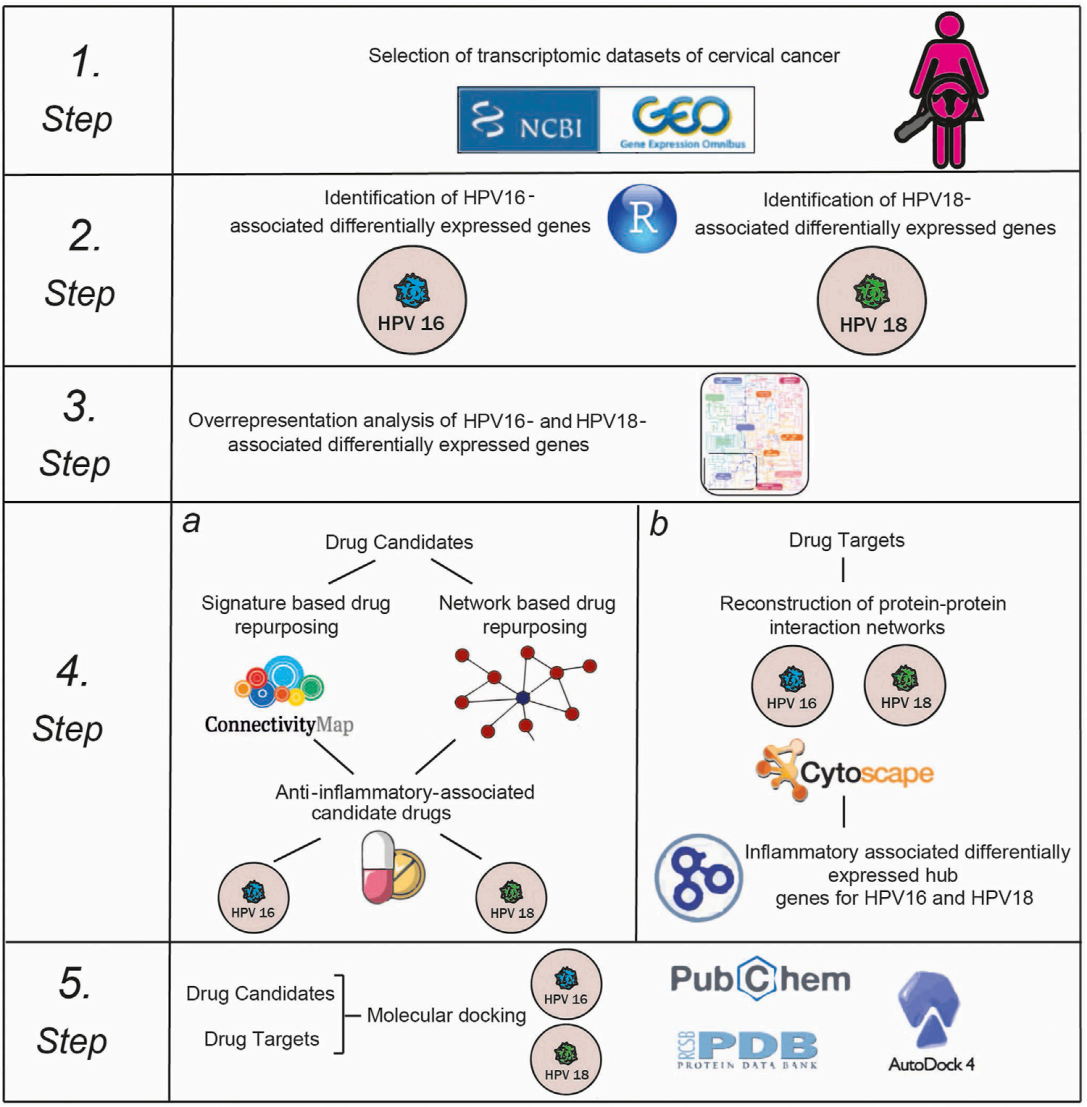
The multi-stage computational approach was employed in the study.

Here, we repositioned anti-inflammatory drug candidates targeting inflammation-associated hub proteins using two different drug repositioning strategies to treat cervical cancer in a subtype-specific (HPV16 and HPV18) manner. To this end, we used a multistep computational approach (Figure 1). First, we performed a meta-analysis of cervical cancer-associated transcriptomic datasets by accounting for subtype differences between samples and identifying differentially expressed genes (DEGs). Using gene signature reversal on HPV16 and HPV18, we performed a signature-based repositioning to identify candidate anti-inflammatory drugs. In addition, we employed a network-based drug repurposing approach. We reconstructed protein-protein interaction networks around HPV16- and HPV18-associated DEGs to identify inflammation-associated hubs. The inflammation-associated drug candidates were evaluated using molecular docking to determine the potential of physical interactions between the anti-inflammatory drug and the inflammation-associated hubs as drug targets. Consequently, our computational study proposed anti-inflammatory drug candidates targeting inflammatory proteins of HPV16 and HPV18 subtypes of cervical cancer.

## Materials and Methods

### Selection of Transcriptomic Datasets

Since, our aim was to reveal the appropriate subtype-specific (i.e., HPV16 and HPV18) personalized drugs for cervical cancer, the transcriptomic datasets were evaluated considering the genotypes of the diseased samples. In addition, to avoid undesirable alterations originating from differences in the microarray platforms used, we ensured that the transcriptome datasets were generated using the same platform. With this in mind, we found a total of five transcriptome datasets that corresponded to the HPV16 or HPV18 genotype whose gene expression measurements were performed using the same platform (Affymetrix microarrays). The five transcriptome datasets were as follows: GSE52903 (Medina-Martinez et al., 2014), GSE39001 (Espinosa et al., 2013), GSE9750 (Scotto et al., 2008), GSE7803 (Zhai et al., 2007) and GSE6791 (Pyeon et al., 2007). High-grade squamous lesions were excluded from GSE7803, healthy endocervical tissue samples were excluded from GSE39001, and only cervical cancer samples were included in the GSE6791 datasets to avoid sample heterogeneity. While all five datasets contained samples belonging to the HPV16 genotype, only three datasets (GSE9750, GSE7803 and GSE6791) contained samples of HPV18. Thus, 111 HPV16-positive diseased samples were compared with 61 controls, while 10 HPV18-positive diseased samples were compared with 39 control samples.

### Identification of Differentially Expressed Genes and Overrepresentation Analysis

This study used a well-established statistical analysis procedure (Kori et al., 2019) to identify DEGs. Briefly, the raw data (stored in. CEL files) of each dataset was normalized by calculating the Robust Multi-Array Average (RMA) expression measure (Bolstad et al., 2003) as implemented in the Affy package (Gautier et al., 2004) of the R/Bioconductor platform (version 4.0.2) (Huber et al., 2015). DEGs were identified from normalized expression values using the Linear Models for Microarray Data (LIMMA) package (Ritchie et al., 2015). The Benjamini–Hochberg method was used to control false discovery rate (FDR). The adjusted *p*-value < 0.05 was used as a cut-off to determine the statistical significance of the DEGs. Fold change was used to determine the regulatory pattern of each DEG (i.e., up- or down-regulation), and at least 1.5-fold change was considered statistically significant. Further analyses were performed with DEGs that shared at least three of the five HPV16 datasets, referred to as “HPV16-associated DEGs,” while analyses were performed with DEGs that shared at least two of the three HPV18 datasets, referred to as “HPV18-associated DEGs.” In this way, the analysis performed with DEGs that occurred in at least 60% of all datasets.

Overrepresentation analyses were performed using ConsensusPathDB (Kamburov et al., 2013) to determine pathways hijacked by HPV16- and HPV18-associated DEGs. KEGG database resources provided by ConsensusPathDB were preferred in the analysis. *p*-values were determined *via* Fisher’s Exact Test, and FDR was applied to control *p*-values. For overrepresentation analyses, an adjusted *p*-value < 0.05 was considered statistically significant.

### Signature-Based Drug Repurposing

To reveal the correlations of gene signatures of drugs, the database CLUE (Subramanian et al., 2017) was used. HPV16- and HPV18-associated DEGs were used as queries and analyzed individually. We filtered our DEG data because we have more than 150 genes as queries, and there is a size limit of 10–150 genes in the CLUE database. For this purpose, we ordered the DEGs according to their fold-change and determined the first 150 genes with the lowest fold-change and the 150 genes with the highest fold-change and used them as down-regulated and up-regulated genes as queries, respectively. For a given query set pair, the database CLUE assigns connectivity scores to the perturbations in the form of Kolmogorov-Smirnov statistics and random permutation tests. The connectivity score ranges from −100 to +100, and the negative score indicates an inverse pattern, meaning that genes that were increased as a result of perturbation treatment are genes that were decreased in the query. By default, drugs with -90 connectivity scores were considered significant.

### Network-Based Drug Repositioning

A web-based transcriptome-driven drug repositioning application tool, geneXpharma (Turanli et al., 2017), was used for network-based drug repositioning analyses. The tool contains gene-drug interactions (obtained from Drug Gene Interaction Database) and gene-disease libraries. The gene-disease library was created by analyzing 118 different transcriptome datasets (corresponding to different 48 diseases) DEGs. Consequently, this tool provides the association of the drug to a DEG (disease dataset) considering hypergeometric distribution function. The gene-drug association library in geneXpharma contains 50,304 gene-drug interactions involving 4344 genes and 11,939 drugs. In network-based drug repositioning analyses, we used HPV16- and HPV18-associated DEGs individually as queries and identified whether the disease and drug candidates interacted with our DEG query lists. We considered drug candidates significant with a hypergeometric *p*-value < 0.01.

### Determination of Anti-Inflammatory Associated Drugs

Anti-inflammatory drugs were identified through an anatomical therapeutic chemical (ATC) classification system and a literature review. Drugs with ATC codes M01A (anti-inflammatory and anti-rheumatic products, non-steroids), H02AB (corticosteroids for systemic use, plain) and N02BA (salicylic acid and derivatives) were selected as anti-inflammatory drugs and obtained from the DrugBank resource (version 5.1.7) (Wishart et al., 2006). The drugs that also have anti-inflammatory activity and have already been reported in the literature were also included in the study. Thus, a total of 127 anti-inflammatory drugs were found.

### Reconstruction and Analysis of Protein-Protein Interaction Networks

The human protein interactome was derived from a previously published study (Cheng et al., 2019) containing 243,603 experimentally confirmed protein-protein interactions (PPIs) among 16,677 unique proteins from five data sources. PPI networks were represented as undirected graphs, with nodes representing proteins and edges representing interactions between proteins. PPI networks were reconstructed individually for HPV16- and HPV18-associated DEGs with their first neighbors and visualized using Cytoscape (v3.5.0) (Shannon et al., 2003). To determine hub proteins (i.e., central proteins), topological analyzes were performed using the Cytohubba plugin (Chin et al., 2014). The degree of a node, representing the number of edges connected to the node, was determined. The top 3% of nodes, ranked by highest degree, were considered hub proteins. The hub proteins that were DEG at the same time were further analyzed.

### Determination of Inflammatory Associated Hubs

To identify the inflammation-associated hub DEGs, we first specified proteins previously associated with inflammation. To this end, proteins classified in the inflammatory response process (GO: 0006954) were screened in QuickGO, a web-based tool for searching Gene Ontology annotations (version 2021-01-08) (Binns et al., 2009). In addition, we screened for inflammation-associated proteins using the keyword “inflammation” in the UniProt portal (The UniProt Consortium, 2019). Thus, a total of 1215 inflammation-associated proteins were found. The culminated hubs were integrated with the generated list of inflammation-associated proteins, and inflammation-associated hub DEGs were identified.

### Molecular Docking Simulations

The three-dimensional (3D) crystal structure of the target proteins (i.e, inflammation-associated hubs) was taken from Protein Data Bank (PDB) (Berman et al., 2002) when available. The structures of the anti-inflammatory drug candidates were obtained from the PubChem database (Kim et al., 2019). Molecular docking analyses were performed using AutoDock Vina software (Trott and Olson, 2010). In the analyses, previously known binding residues of target proteins were used for docking drug candidates. Binding affinities (kcal/mol) were reported to determine binding significance after molecular docking. In addition, known target protein inhibitors from the literature were sought as positive controls, and molecular docking simulations were also used for these inhibitors.

## Results

### Identification of Differentially Expressed Genes

As a result of the analysis, thousands of individual DEGs were identified according to the criteria we established (adjusted *p*-value <0.05 and log2FC >|0.58|). The expression patterns (up- or down-regulation) of the culminated DEGs for five HPV16 transcriptome datasets were numerically almost identical. Namely, a total of 51% of DEGs were up-regulated. In addition, the expression patterns of the culminated DEGs for HPV18 tended to be up-regulated (56%).

**FIGURE 2:**
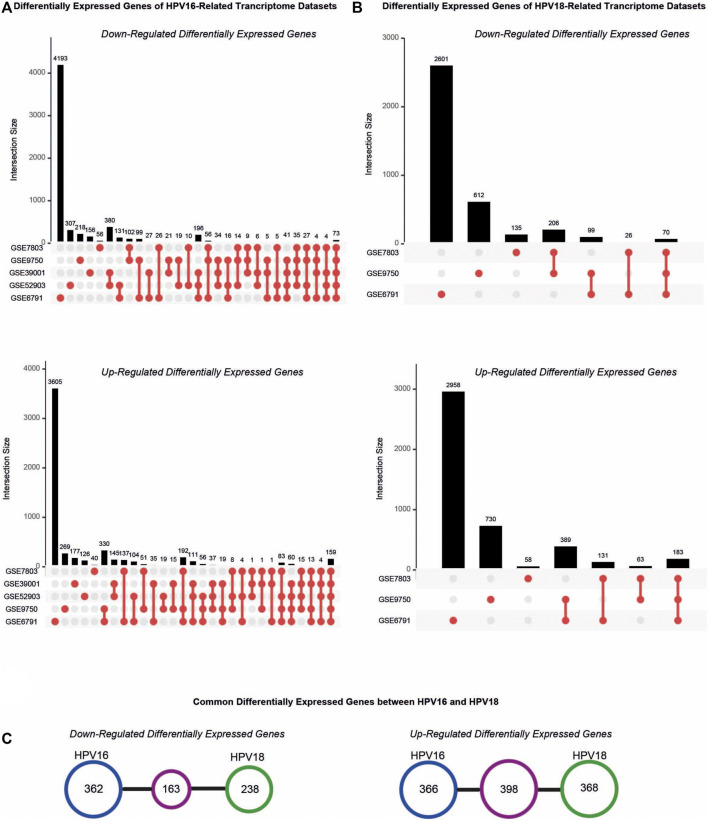
The distribution of differentially expressed genes (DEGs) in the cervical cancer transcriptome datasets. **(A)** The upset plots represent DEGs in the transcriptome datasets that compromised HPV16 cervical cancer samples. **(B)** The upset plots represent DEGs in the transcriptome datasets that compromised HPV18 cervical cancer samples. **(C)** The diagrams represent the common DEGs in the HPV16- and HPV18 transcriptome datasets.

The resulting DEGs were comparatively analyzed considering their subtypes (i.e. HPV16 or HPV18). Further analyses were performed for HPV16 DEGs culminating in at least three datasets, referred to as “HPV16-associated DEGs” and for HPV18 DEGs common in at least two datasets, referred to as “HPV18-associated DEGs”. A total of 1289 (525 down- and 764 up-regulated) HPV16-associated DEGs and 1167 (401 down- and 766 up-regulated) HPV18-associated DEGs were identified (Figures 2A,B). In addition, 163 down-regulated and 398 up-regulated genes culminated between the HPV16- and HPV18-associated DEGs (Figure 2C).

**FIGURE 3:**
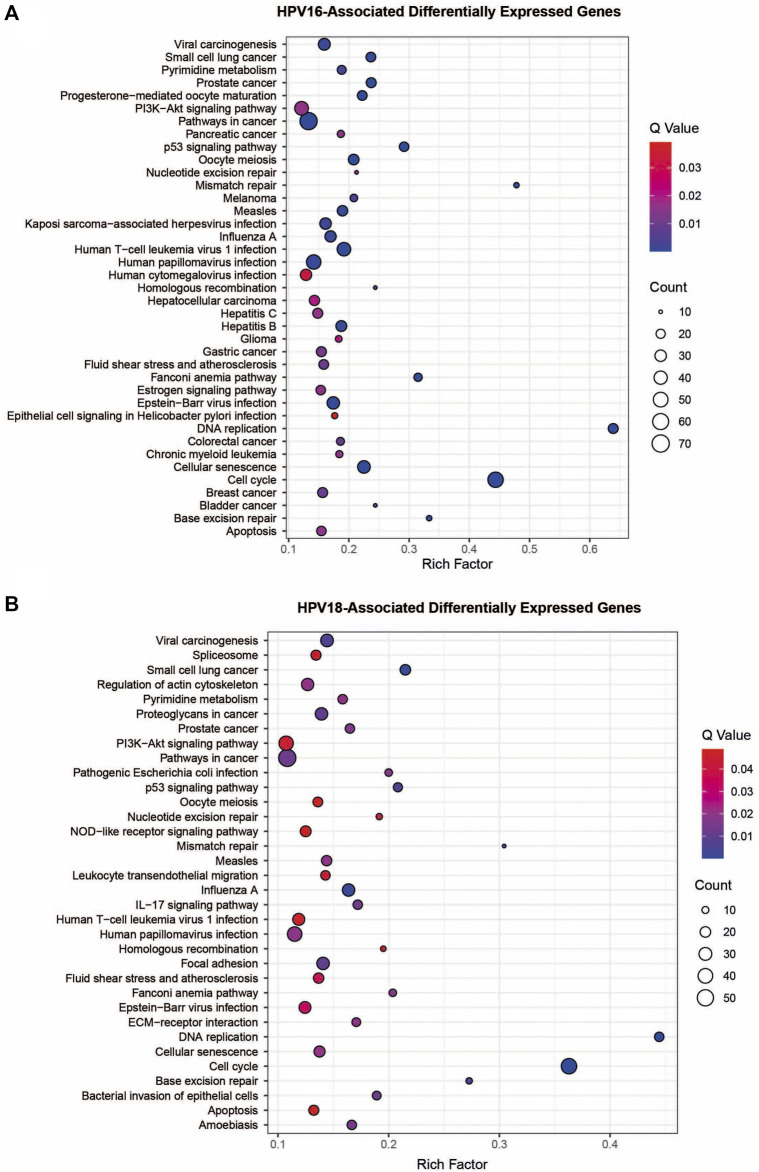
The pathway overrepresentation analysis of HPV16- and HPV18-associated differentially expressed genes (DEGs). **(A)** A plot representing overrepresentation analysis of HPV16- associated DEGs. **(B)** A plot representing overrepresentation analysis of HPV18- associated DEGs.

Overrepresentation analyses were performed to gain further biological insight into HPV16- and HPV18-associated DEGs. Both HPV16- and HPV18-associated DEGs were enriched in vital processes such as cell cycle, DNA replication, cellular senescence, p53 signaling pathway, and apoptosis. Furthermore, they are associated with infectious and infection-associated disease pathways, including human T-cell leukaemia virus 1 infection, Epstein-Barr virus infection, measles, HPV infection, influenza A, and viral carcinogenesis. In addition to cancer pathways, including colorectal cancer, bladder cancer, breast cancer, gastric cancer, pancreatic cancer, hepatocellular carcinoma, and infections such as hepatitis B/C and Kaposi’s sarcoma-associated herpesvirus infection were specific pathways for HPV16-associated DEGs. HPV18-associated DEGs were specifically enriched with pathways such as focal adhesion, pathogenic *E. coli* infection, and ECM-receptor interaction (Figures 3A,B).

### Drug Repurposing With Two Different Strategies

To investigate the applicability of anti-inflammatory drug therapies specific to subtypes of cervical cancer (HPV16 and HPV18), a drug repurposing analysis was performed using two different strategies (i.e., signature-based and network-based).

**FIGURE 4:**
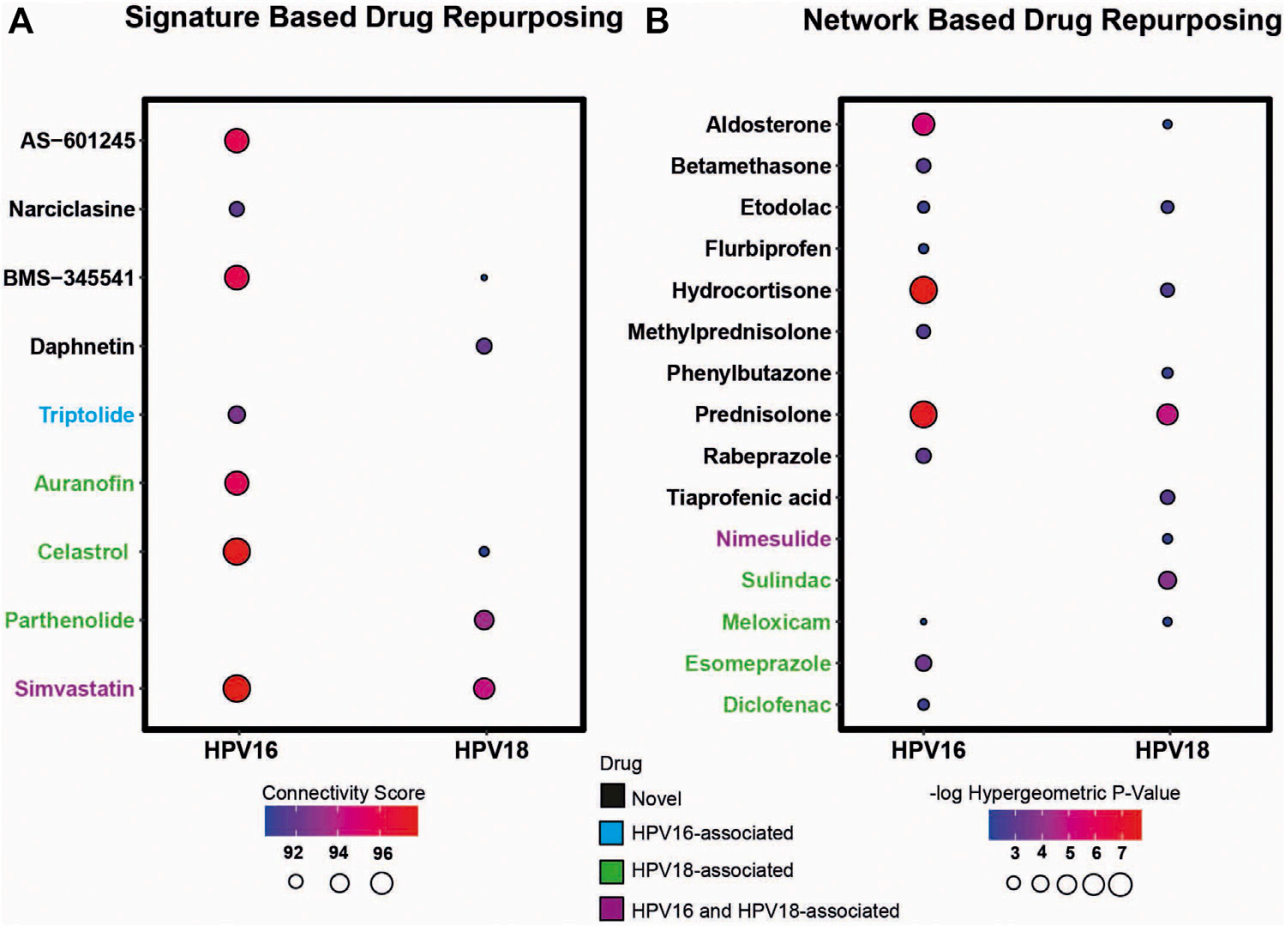
The candidate drug targets that were revealed based on two different drug repurposing strategies (i.e. signature based and network based strategy). **(A)** The bubble plot indicates the drugs that resulted according to the signature-based drug repurposing strategy. **(B)** The bubble plot indicates the drugs that resulted according to network-based drug repurposing strategy. The drugs colored in black mean that the culminated drugs have not been associated with HPV16 or HPV18 subtypes of cervical cancer previously and novel. The drugs colored in blue, green, and purple means the culminated drugs have been associated with HPV16, HPV18 and with both subtypes of cervical cancer previously.

First, transcriptomic gene signatures corresponding to HPV16- and HPV18-associated DEGs were used as input to query the CLUE database (Subramanian et al., 2017) to assess drug-induced expression profiles. Drugs with a connectivity score < −90 were considered significant in the following analysis. A total of 79 drug candidates were identified for the HPV16 subtype (Supplementary Table S1). Among them, seven drugs were defined as anti-inflammatory associated drugs when integrated into our list of anti-inflammatory drugs. In addition, by using HPV18-associated DEGs as input, a total of 60 drug candidates were specified (Supplementary Table S2), of which five drugs were available as anti-inflammatory drugs. Ultimately, 3 anti-inflammatory associated drugs (BMS-345541, Celastrol—also known as Triptin - and Simvastatin) were found in two HPV subtypes according to their connectivity score significance. Additionally, 4 (AS -601245, auranofin, narciclasin, and triptolide) and 2 (daphnetin and parthenolide) anti-inflammatory drugs were found to be specific to HPV16 and HPV18 subtypes, respectively (Figure 4A).

As a second strategy, the transcriptome-guided drug repositioning tool, geneXpharma (Turanli et al., 2017), was applied to evaluate whether the diseases and drugs interacting with our DEG query list were specific to our studied two HPV subtypes. The tool assigns a hypergeometric *p*-value to each drug, and drugs with a hypergeometric *p*-value < 0.01 were accepted as significant in the analysis. As a result, we found a total of 611 different drugs for the HPV 16 subtype (Supplementary Table S3), of which 11 had an anti-inflammatory origin. Moreover, out of 254 drug candidates for the HPV18 subtype (Supplementary Table S4), 9 drug candidates belonged to the anti-inflammatory class. Overall, anti-inflammatory drugs such as aldosterone, etodolac, hydrocortisone, meloxicam and prednisolone culminated into two different subtypes. Betamethasone, diclofenac, esomeprazole, flurbiprofen, methylprednisolone, and rabeprazole specifically induced the only HPV16 subtype, whereas nimesulide, phenylbutazone, sulindac, and tiaprofenic acid specifically induced the only HPV18 subtype (Figure 4B).

Following the two strategies employed, we manually reviewed the listed candidate anti-inflammatory drugs in PubMed to determine if the drugs had been previously associated with cervical cancer. The literature review showed that some drugs have been previously associated with cervical cancer. The drugs auranofin (You et al., 2015), celastrol (Hu et al., 2013), diclofenac (Al-Nimer et al., 2015), esomeprazole (Jumaa et al., 2020), meloxicam (Dyakova et al., 2015), parthenolide (Jeyamohan et al., 2016) and sulindac (Karl et al., 2007) were previously associated with cervical cancer HPV18 subtype. Triptolide (Qin et al., 2018) was associated with the HPV16 subtype, and drugs such as nimesulide (Soriano-Hernandez et al., 2015) and simvastatin (Pan et al., 2020) were previously associated with both cervical cancer subtypes. We considered these results as positive controls for subsequent analyzes. The remaining 6 anti-inflammatory drugs for the HPV16 subtype, 2 anti-inflammatory drugs for the HPV18 subtype, and 5 anti-inflammatory drug candidates for both subtypes were novel candidates. Therefore, they were considered as candidate drugs and analyzed further (Figure 4).

### Reconstruction of Protein-Protein Interaction Networks and Discovery of Inflammatory Associated Hubs

**FIGURE 5:**
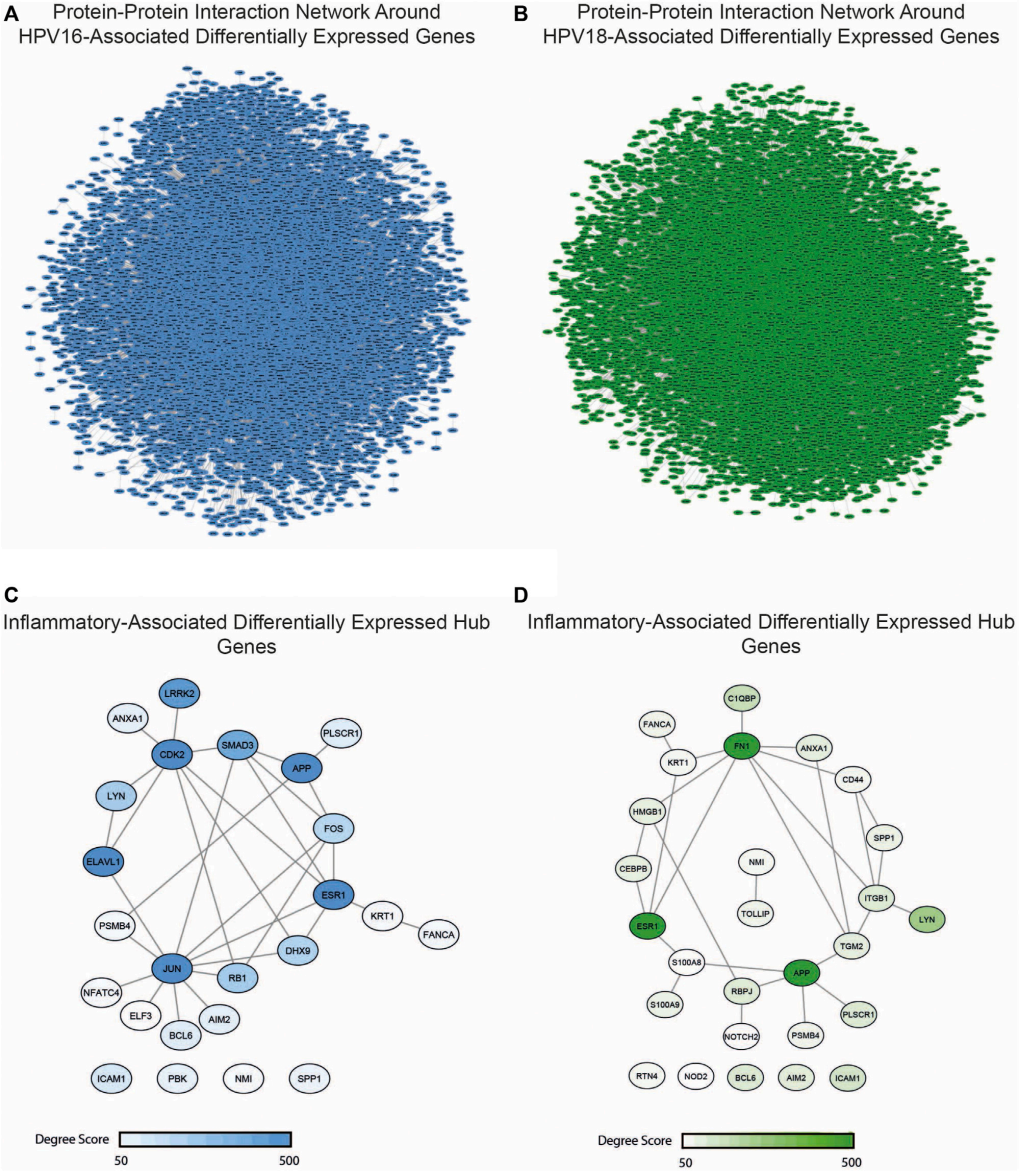
The reconstructed protein-protein interaction (PPI) network around the HPV16- and HPV18-associated differentially expressed genes (DEGs) and inflammatory-associated hub DEGs. **(A)** The reconstructed PPI network around HPV16-associated DEGs **(B)** The reconstructed PPI network around HPV16-associated DEGs **(C)** The anti-inflammatory and HPV16 associated hub DEGs which colored according to degree score significance **(D)** The anti-inflammatory and HPV18 associated hub DEGs which colored according to degree score significance.

The study of diseases using PPI networks contributes to elucidating interrelationships between proteins and is crucial for uncovering new insights into pathogenesis. Since hubs organize the global structure of the network and play a central role, they represent potential drug candidates. In this study, we first reconstructed PPI interactions to uncover target candidates. The reconstructed PPI interaction network around HPV16-associated DEGs consisted of 10360 nodes with 50519 edges, while the interaction network in HPV18-associated DEGs consisted of 12379 nodes with 57331 edges (Figures 5A,B). After reconstructing the interaction network, the degree of nodes in the networks was determined. A total of 257 hubs and 306 hubs that were simultaneously DEG were found for HPV16 and HPV18 subtypes, respectively. The DEG hubs were integrated with the constructed list of inflammation-associated proteins, and it was found that of the 257 hub DEGs, 24 were associated with inflammation and of the 306 hub DEGs, 27 were associated with inflammation (Figures 5C,D). The culminated inflammation-associated hub DEGs were considered as anti-inflammatory drug targets and further analyzed with docking simulations.

### Molecular Docking Simulations

**FIGURE 6:**
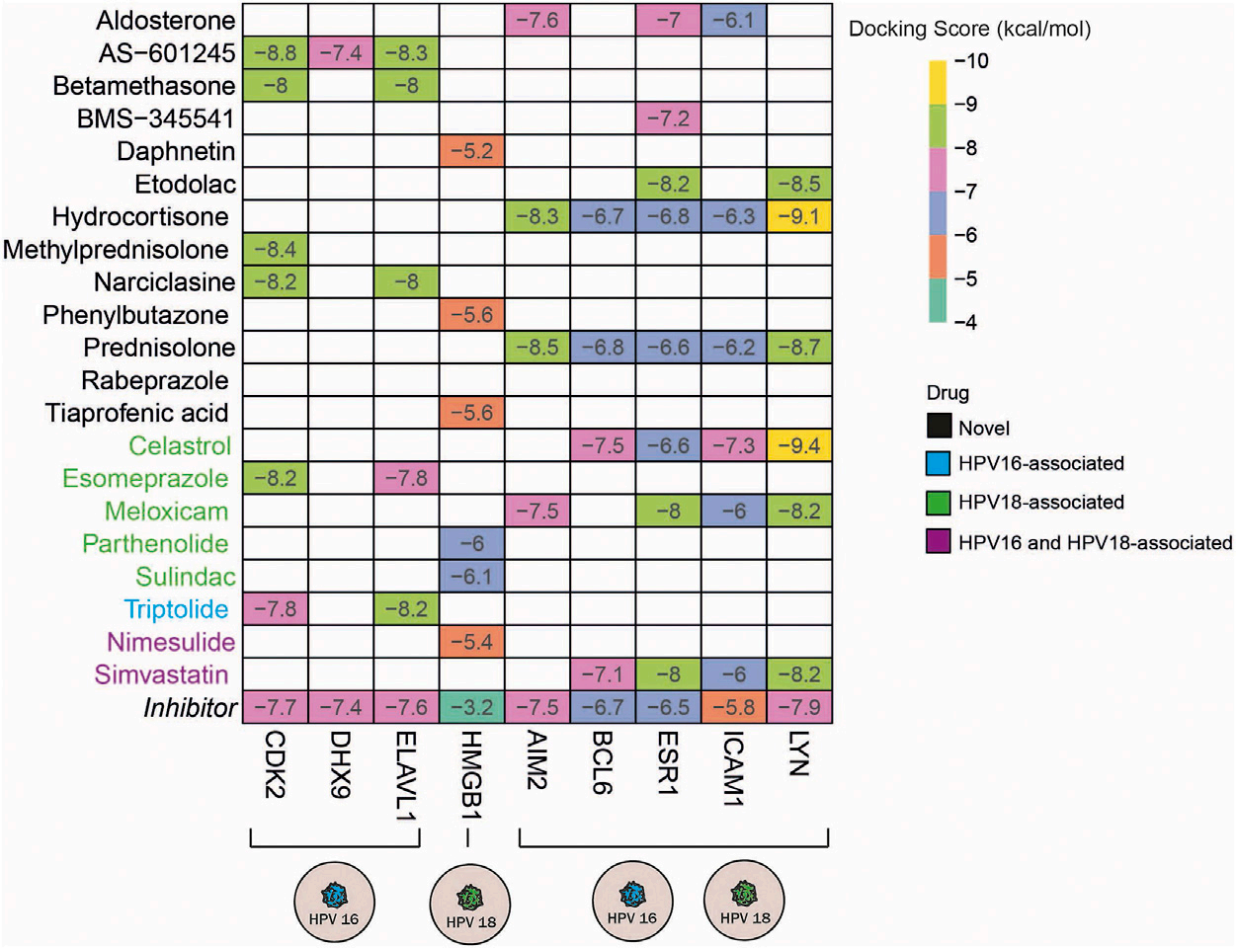
The heatmap indicates the docking scores of the repurposed drugs. Only values with a lower docking score than the drug’s inhibitor are represented. The drugs colored in blue, green, and purple means the culminated drugs have been associated with HPV16, HPV18, HPV16, and 18 subtypes of cervical cancer previously.

Molecular docking simulates the binding affinity of a drug in the 3D structure of a drug target. Overall, we performed molecular docking simulations to evaluate whether the candidate anti-inflammatory drugs target inflammation-associated hub DEGs. To this end, we first screened the 3D structure of inflammation-associated hub DEGs and found the suitable 3D structures for 15 out of 38 targets. In addition, the 3D structure for each drug candidate was determined from the corresponding database and included in the molecular docking simulations. To specify the significance of the docking score, we screened known inhibitors of the 15 targets and used them as positive controls (Supplementary Table S5). Following the literature search, we found inhibitors for 13 of the 15 targets and performed docking simulations for them as well. As a result of the molecular docking simulations, we identified the following drugs AS -601245 (targets CDK2, DHX9, and ELAV1), betamethasone, narciclasin (all 3 drugs target CDK2 and ELAV1), and methylprednisolone (targets CDK2) as HPV16 subtype-specific novel anti-inflammatory drug candidates. The drugs, including daphnetin, phenylbutazone, and tiaprofenoic acid, all targeting the inflammation-associated protein HMGB1, were found to be specific for the HPV18 subtype. Finally, the anti-inflammatory drugs aldosterone (targets AIM2, ESR1 and ICAM1), BMS-345541 (targets ESR1), etodolac (targets ESR1 and LYN), hydrocortisone (targets AIM2, BCL6, ESR1, ICAM1, and LYN), and prednisolone (targets AIM2, BCL6, ESR1, ICAM1, and LYN) yielded significant and novel results for both HPV16 and HPV18 subtypes (Figure 6).

## Discussion

Cervical cancer is one of the leading causes of death in women, and yet the treatment strategies used have not been adequate and specific for cervical cancer. Therefore, these results clearly demonstrate the need to develop more effective prevention and/or treatment strategies. In this study, we proposed new anti-inflammatory drugs for HPV16 and HPV18 subtypes of cervical cancer that simultaneously target inflammation-associated hub DEGs. In addition, we also found drugs that were already associated with the subtypes we studied, further strengthening our confidence in our observations.

Our approach differs from previous drug repositioning studies. To our knowledge, this was the first study in the literature under three aspects: 1) the repositioned drugs and their targets were all associated with anti-inflammatory agents, 2) the drug repurposing approach was used with two different strategies, and 3) the differences between HPV16 and HPV18 subtypes were considered in the analysis.

Inflammation is linked to cancer and plays an important role in tumor growth and progression through epidemiological studies (Greten and Grivennikov, 2019). As a result, drug repositioning efforts focusing on inflammation and the chemicals involved in the inflammatory process are paying an attention for an effective cancer preventive and treatment method (Turanli et al., 2018). Several clinical investigations have indicated that anti-inflammatory medicines, such as non-steroidal anti-inflammatory drugs (NSAIDs), can disrupt the tumor microenvironment by slowing cell migration, boosting apoptosis, and improving chemosensitivity (Zappavigna et al., 2020). Due to the link between inflammation and cancer, repositioning known anti-inflammatory medicines used in cancer therapy and their mechanisms of action, as well as the usage of novel anti-inflammatory compounds with anticancer efficacy become more promising for the cancers triggered by persistent infections.

As persistent infections and/or chronic inflammation are the main reasons for cancer development, persistent HPV infection with high risk is undoubtedly crucial for cervical cancer progression. In addition to persistent infection, studies have clearly shown that long-term chronic inflammation contributes to the development of cervical cancer (Fernandes et al., 2015). Given all these information, it is reasonable to assume that preventing inflammation may be a beneficial approach to the prevention and/or treatment of cervical cancer.

The main indications for anti-inflammatory drugs are fever, pain, and inflammation. However, many anti-inflammatory drugs have properties similar to neoplastic agents in that they promote apoptosis, inhibit angiogenesis, and enhance the immune response (Wong, 2019). Therefore, the use of anti-inflammatory drugs in cervical cancer is like “killing two birds with one stone.” They have both anti-inflammatory and anti-cancer effects in the treatment of cervical cancer. Therefore, this is the main reason for choosing anti-inflammatory drugs and targets as targets in this study.

According to GLOBOCAN data on cervical cancer, low-income countries have higher mortality and higher incidence (Sung et al., 2021). Cervical cancer is the most common disease among women in developing countries such as Africa, Asia, and South America, which could be due to lack of screening programs, insufficient funding, and inadequate access to health care or even anti-vaccination campaigns. The prevalence of cervical cancer in developed countries has declined thanks to improved health care and widespread availability of preventive HPV vaccines, which is an important step in preventing HPV-related malignancies. On the other hand, prophylactic vaccines have proven useful only in healthy individuals and cannot treat or prevent an infection that has already broken out. Recurrence is possible with current treatments such as surgical resection, radiation, or chemotherapy, which do not specifically target the carcinogenic properties of HPV. In addition, most of these procedures can damage normal tissues and have potential adverse effects, such as bleeding, which can make patients uncomfortable and affect quality of life. This is another reason to focus on HPV-related carcinogenesis, which can be treated with anti-inflammatory drugs that are cheap, easy to obtain, and associated with tolerable side effects (Gomes et al., 2021).

We used gene signature and network-based drug repurposing strategies to identify drug candidates. The typical starting point of both strategies was gene signatures derived from cervical cancer transcriptomic data. When repositioning drugs based on gene signatures, we used the effect of reverse expression of the disease state, as in many studies (Turanli et al., 2019; Beklen et al., 2020). However, we also reconstructed combinations of gene signature and disease state in network-based drug repurposing. We anticipate that by combining both strategies, reliable candidates can be found for further experimental studies.

Although HPV16 and HPV18 are the two viruses responsible for most cases of cervical cancer, they represent different HPV subtypes. The HPV subtypes may have different biological mechanisms and affect cancer progression differently. This natural variability of cancer promotes the development of personalized medicine. The password of personalized medicine stands for the right drug, for the right patient, at the right time, and at the right dose (Sadeè and Dai, 2005). Although the HPV16 and HPV18 is the most encountered subtypes of the cervical cancer we focus on these two subtypes and offer drugs that special for these two subtypes. Besides, we proposed drug candidates that can be useful for both sub-types considering the underdeveloped countries where such HPV typing is not routinely performed. Based on our analysis, we identified promising drug candidates for the treatment of cervical cancer subtypes and proposed candidates for further experimental studies. By integrating transcriptome datasets with 2 different drug repurposing strategies, we identified 4 novel anti-inflammatory HPV16-specific drug candidates (AS -601245, betamethasone, narciclin, and methylciclin) and validated narciclasin and methylprednisolone by *in silico* analysis. We also identified 3 new HPV18-specific drug candidates (daphnetin, phenylbutazone, and tiaprofenic acid) and 5 drug candidates (aldosterone, BMS-345541, etodolac, hydrocortisone, and prednisolone) for the treatment of both subtypes. These five candidate drugs can be highlighted particularly for underdeveloped countries where cervical cancer is very common and subtyping is not routinely done. We reported valuable data for further experimental and clinical efforts, as the proposed anti-inflammatory drug candidates can be used as therapeutics for the prevention and/or treatment of cervical cancer. The major limitation of the study is the lack of experimental validations of the identified anti-inflammatory drugs on the cervical cancer tissue samples or cell lines. Future *in vitro* studies need to be performed to investigate the effects of the identified drug candidates on cell viability, proliferation, and migration. Moreover, the mechanism of action of these molecules needs to be studied experimentally to elucidate their effects on important molecular signaling pathways such as cell death and cell replication. In addition, the proposed drug candidates can not only be considered as single agent candidates, but can also be used in combination, so that the effect of combination therapy can also be validated by *in vitro* studies.

## Data Availability

The datasets presented in this study can be found in online repositories. The names of the repository/repositories and accession number(s) can be found in the article/Supplementary Material.

## Abbreviations

ATC, anatomical therapeutic chemical; DEG, differentially expressed genes; FDR, false discovery rate; HPV, Human Papillomavirus; PPI, protein-protein interaction.
